# Labor markets during COVID-19: gaps and challenges in Latin America

**DOI:** 10.3389/fsoc.2024.1372404

**Published:** 2024-09-25

**Authors:** Oscar A. Martínez-Martínez, Javier Reyes-Martínez, Andrés Iván Mideros Mora, Andrea Carolina Sánchez Pilco, Camila Lucia Rodríguez Salme

**Affiliations:** ^1^Departamento de Ciencias Sociales y Políticas, Universidad Iberoamericana, Mexico City, Mexico; ^2^División de Administración Pública, Centro de Investigación y Docencia Económicas, Mexico City, Mexico; ^3^Facultad de Economía, Pontificia Universidad Católica del Ecuador, Quito, Ecuador; ^4^Pontificia Universidad Católica del Ecuador, Instituto de Investigaciones Económicas, Quito, Ecuador

**Keywords:** labor formality, COVID-19, gender, economic sectors, Latin America

## Abstract

**Introduction:**

The effects of the COVID-19 pandemic on Latin American labor markets continue to be quantified, to identify the social and economic impacts that this pandemic had, and to design more efficient public policies that would protect the most vulnerable groups. For this reason, the research question was as follows: what were the changes in the labor formality rates before and two years after the main contingency measures of the COVID-19 pandemic were implemented?

**Methods:**

Using data from Argentina, Bolivia, Chile, Colombia, Ecuador, Guatemala, Mexico, Peru, and Uruguay, the formality rate (τ) was analyzed, which was calculated using a weighted average between the formal employment rates of the number (i) of economic sectors (p) in a specific period (t).

**Results:**

The results suggest that the weighted labor formality rate increased in the countries of the region. These changes in formality could be the result of greater capital accumulation, the integration of productive processes, the integration of commercialization processes, and differentiated fiscal stimuli (i.e., the intrasectoral aspect), but it was not due to the displacement of workers from highly informal economic sectors to more formalized sectors (i.e., the intersectoral component).

**Discussion:**

The findings emphasized the precarious situation of women in the region, regardless of the country, particularly in Ecuador, Peru, Bolivia, and Argentina. These findings suggest the need to design public policies that reverse the current situation of the labor market and prevent future economic shocks, with special emphasis on the informal sector and women.

## 1 Introduction

On March 11, 2020, the World Health Organization (WHO) declared COVID-19 a pandemic, noting that all governments should take strict measures to address this global problem (WHO, 2020). COVID-19 had immediate effects on various economic and social factors, especially activities that involved physical proximity (ECLAC, 2020). Therefore, restrictions that were imposed to stop the transmission of the virus impacted production, private consumption, and gross fixed investment (Banxico, 2021). These measures generated concern regarding their consequences, such as a potential exogenous shock that would lead to an economic crisis for productivity and employment (Moreno and Cuellar, 2021). Most worldwide economies limited their activities, which affected the labor market (SHCP, 2021) and generated a series of problems at a global level, especially in Latin America.

Before the pandemic, it was estimated that the Latin American region would grow a maximum of 1.3% in 2020; however, the effects of the pandemic led to a decrease in the Gross Domestic Product (GDP) by 1.8% and an economic contraction between 3 and 4% (ECLAC, 2020). Furthermore, this region has historically experienced high unemployment rates and constant job insecurity (ECLAC and ILO, 2021). Before the pandemic, the labor formality rate was very low, and there was little mobility in the labor market at the structural level, with most of the labor force being classified in the informal sector (Martínez-Martínez et al., 2022). Between 2018 and 2019, the informal sector rate fluctuated by 24.2% (LAPOP, 2020). This was due to the recurring economic contractions in the region, which led to the loss of formal work during economic cycles (ILO, 2020) and an increase in informal labor for various population groups (Lara and Rojas, 2021). Additionally, medium and large companies had more incentives to hire informal workers (Busso et al., 2012) because labor benefits reduced the incentives to hire formal workers, leading to mutual agreements with the workers in some cases (Martínez and Torres, 2016).

The conditions that arose at the start of the pandemic led to a deterioration in the labor market in the region (ECLAC and ILO, 2021), resulting in an increase in unemployment, and reduction in working hours. Studies on the effects of COVID-19 on labor markets have shown that the pandemic generated a massive increase in unemployment (Kesar et al., 2021), which affected all economic sectors (Ruiz, 2020). The International Labor Organization (ILO, 2020) found that both formal and informal employment experienced contractions. However, the informal sector experienced the greatest repercussions because most informal workers had a decrease in income due to confinement/lockdown, and they did not have access to unemployment insurance (Pitoyo et al., 2020, 2021).

In previous crises, informality was a mechanism of protection for those who had lost their jobs in the formal sector (Mapp and Moore, 2015). However, people who lost their jobs during the COVID-19 pandemic faced restrictions, such as confinement (especially during the first 2 years), where they could not leave their homes to look for formal or informal work. Thus, 2020 was an atypical year in terms of job creation, because the pandemic demanded measures that restricted economic activity, and part of the population was left without a source of income (Barrera, 2022). According to the Economic Commission for Latin America and the Caribbean ECLAC and ILO (2021b), the COVID-19 pandemic caused a significant decrease in the informal sector rate, but these declines did not indicate progress in labor formalization but rather indicated that informal activities were more affected by the COVID-19 measures. Individuals without an income due to unemployment experienced vulnerabilities that were associated with non-compliance with human rights (Reyes-Martínez and Andrade-Guzmán, 2021) and poverty (ILO, 2013).

When a lack of employment impacts socially disadvantaged groups, a double vulnerability is experienced, as is the case for women. Before the pandemic, women’s main option for work in the Latin American region was informal or lower-quality employment (ILO, 2013), regardless of individual and household characteristics (Acemoglu, 2001), which indicates inequality in rights and job opportunities (Anwary, 2017; Domínguez-Amorós et al., 2019). During the pandemic, the female labor sector experienced high unemployment rates, late reentry, decreased labor force participation, and decreased rates of hiring, particularly in positions of high responsibility (Moreno and Cuellar, 2021; WEF, 2021). For these reasons, the gender gap in the labor market participation increased, mainly due to the partial return of school activities, limited access to caregiving services, and slow recovery in sectors, such as commerce, services, and paid domestic work, where informality is prevalent (Zamudio et al., 2022). Women with children have a greater probability of participating in the informal sector due to the barriers to entry that hinder their access to formal jobs (Cuevas et al., 2016). In addition, women conducted more domestic work and unpaid care during the pandemic because of the closure of schools and the suspension of support services (Zamudio et al., 2022). Moreover, women living in poverty have less availability for jobs because more of their time is taken up by domestic chores (Gutiérrez et al., 2020). For this reason, the ECLAC (2021) stated that the pandemic crisis caused a setback in decreasing the gender inequality gap in employment.

The problems that were presented above led us to this research question: “What were the labor formality rates before the main COVID-19 pandemic contingency measures were implemented and 2 years afterward?” This question was analyzed by gender and economic sector to determine where the biggest disparities exist in Latin America. This region was integrated for our study by Argentina, Bolivia, Chile, Colombia, Ecuador, Guatemala, Mexico, Peru, and Uruguay. This study addressed the gaps in the literature by analyzing the region as a whole and identifying changes in the labor formality rates before and after the COVID-19 pandemic, which was defined as the proportion of the workforce that had access to social security (Contreras and Lacayo, 2022).

Although the COVID-19 pandemic was declared to have ended on May 5, 2023 by the WHO, its effects on Latin American labor markets still need to be quantified, for that reason we attempted to explore this aspect. In light of this, the objective of this study was to analyze the composition of the labor formality rates before and 2 years after the main contingency measures of the COVID-19 pandemic were implemented in Latin America and identify gaps by gender and economic sector. The findings of this study are especially important because the pandemic led to multiple social and political (Baker et al., 2020) consequences that continue to be a major challenge for public policy and policymakers in Latin America, particularly because the conditions before the pandemic were very heterogeneous. In addition, the effect of COVID-19 was different in each country in our study due to the different policies undertaken by governments, which ranged from support for workers and companies to only maintaining the social programs that were already in place before the pandemic. To name a few, while Belize and Brazil applied the highest percentages of GDP to confront COVID-19 through social programs and different government actions, in other countries such as Ecuador and Mexico, they did not channel more resources to extraordinary programs (ECLAC, 2021) maintaining the pre-pandemic social policy. Hence, the implications of the study can provide guidelines on the design of future policies as well as provide suggestions to address gender gaps in the labor markets in Latin America and beyond.

## 2 Data collection methods and analysis techniques

The purpose of this study was to analyze the composition of the labor formality rates before the main contingency measures of the COVID-19 pandemic were implemented and 2 years afterward in Latin America. This region was selected as the focus of this study due to its complex economic and social parameters, and, according to the ECLAC (2021), it was characterized by almost zero growth prior to the crisis and an economic contraction in 2020 and the weakening of its welfare and social protection systems.

We conducted a secondary data analysis using several datasets. Datasets were selected from countries that met the following criteria: (a) having a wave of data collection that occurred during the first quarter of 2020 (or within a few months), (b) having a wave that was collected during the last quarter of 2021 (or within a few months); and (c) including all the required variables for the analysis (see the Variable subsection below) and having public availability of data.^1^ Therefore, the analysis included datasets from Argentina, Bolivia, Chile, Colombia, Ecuador, Guatemala, Mexico, Peru, and Uruguay. The datasets and their characteristics are presented in Table 1, by country.^2^

**Table 1:** Datasets by country.

Country	Dataset	Sampling design	*N* (first wave)	*N* (second wave)
Argentina	The Permanent Household Survey (EPH, in Spanish)	Probabilistic and two-stage sampling.	51,643 (2020)	50,154 (2021)
Bolivia	The Continuous Employment Survey (ECE, in Spanish)	Probabilistic, stratified, by conglomerates, and two-stage.	8,243 (2020)	11,190 (2021)
Chile	The National Employment Survey (ENE, in Spanish)	Probabilistic, stratified, and two-stage (Instituto Nacional de Estadística de Chile, 2022)	87,842 (2020)	100,433 (2021)
Colombia	The Great Integrated Household Survey (GEIH, in Spanish)	Probabilistic, stratified, cluster, and multistage sample design.	26,595 (2020)	23,744 (2021)
Ecuador	The National Survey of Employment, Unemployment, and Underemployment (ENEMDU, in Spanish)	Probabilistic, stratified, and two-stage.	28,999 (2019)	44,274 (2021)
Guatemala	The National Employment and Income Survey (ENEI, in Spanish)	Master Sampling Framework (MMM) and population projections, based on the XII National Population Census and VII Housing Census of 2018 (see, e.g., INE, 2022)	22,977 (2020)	24,319 (2021)
Mexico	The National Survey of Occupation and Employment (ENOE, in Spanish)	Probabilistic, two-stage, stratified, and by conglomerates (INEGI, s.f.).	417,783 (2020)	434,826 (2021)
Peru	The National Household Survey (ENAHO)	Probabilistic, area, stratified, multistage, and independent sample [Instituto Nacional de Estadística e Informática (INEI, 2021)]	4,763 (2020)	5,086 (2021)
Uruguay	The Continuous Household Survey (ECH, in Spanish)	Random sampling design with two selection stages. The first one is based on the choice of primary sampling units (UPM) that correspond to conglomerates of blocks termed census zones. The second stage corresponds to the extraction of a random sample of dwellings from each UPM [Instituto Nacional de Estadística Uruguay (INE, 2022)]	3,904 (2019)	10,710 (2021)

*N*, Number of observations (year in parentheses). Source: Authors’ elaboration from multiple datasets.

Concerning Argentina, we employ data from the Permanent Household Survey (EPH, in Spanish). EPH is representative at the national level and uses a two-stage and probabilistic sampling. Households are randomly selected in two selection stages. In the first stage, and within each agglomeration, several areas are chosen. In turn, it is performed a random selection of homes from the households in the area. Individuals in these dwellings will be surveyed (see, e.g., Almeida, 2015). In the case of Bolivia, we use the Continuous Employment Survey (ECE, in Spanish). The type of sampling of the ECE is probabilistic, stratified, by conglomerate, and two-stage. It includes geographic coverage over the nine departments and the urban and rural areas Instituto Nacional de Estadística (INE).

Regarding Chile, we use data from the National Employment Survey (ENE, in Spanish). ENE is a representative study at the national, urban national, rural national, and regional level, as well as the metropolitan area of the 16 regions and the rural area of the regions of Coquimbo, Valparaíso, O’Higgins, Maule, Biobío, La Araucanía, Los Lagos, Metropolitana, Los Ríos, and Ñuble. The sample is probabilistic, stratified, and two-stage, where the sampling strata correspond to the combination “Geographic Stratum - Socioeconomic Strata” (Instituto Nacional de Estadísticas de Chile, 2022). In the case of Colombia, the Great Integrated Household Survey (GEIH, in Spanish) has a probabilistic, stratified, cluster, and multistage sample design. The sampling is divided into 2 sub-universes, the first corresponds to the 24 cities within the metropolitan areas and the second refers to the rest of the country made up of municipal capitals [Departamento Administrativo Nacional de Estadística (DANE, 2020)].

Concerning Ecuador, we employ the National Survey of Employment, Unemployment, and Underemployment (ENEMDU, in Spanish). Its sample design is probabilistic, stratified, and two-stage. The study domains of the quarterly ENEMDU are national, rural, and urban areas, and five metropolitan areas (Quito, Guayaquil, Cuenca, Machala, and Ambato) [Instituto Nacional de Estadítica y Censos (INEC, 2021)]. Regarding Guatemala, we use the National Employment and Income Survey (ENEI, in Spanish). The ENEI is representative at the national level, and at the urban and rural level, too. It uses the Master Sampling Framework (MMM) and population projections, based on the XII National Population Census and VII Housing Census of 2018 (see, e.g., INE, 2022).

In the case of Mexico, we employed data from the National Survey of Occupation and Employment (ENOE, in Spanish). The ENOE is representative at the national and state levels, as well as in some selected urban areas. The study comprises more than 126 thousand households. Sampling is probabilistic, two-stage, stratified, and by conglomerates. Selection of the sample is carried out independently by state, area, and stratum (INEGI, s.f.). Regarding Peru, we use the National Household Survey (ENAHO). This study has a probabilistic, area, stratified, multistage, and independent sample [Instituto Nacional de Estadística e Informática (INEI, 2021)].

Concerning Uruguay, the Continuous Household Survey (ECH, in Spanish) uses a random sampling design with two selection stages. The first one is based on the choice of primary sampling units (UPM) that correspond to conglomerates of blocks termed census zones. The second stage corresponds to the extraction of a random sample of dwellings from each UPM [Instituto Nacional de Estadística Uruguay (INE, 2022)].

The labor informality concept was the main variable in this study. According to the ILO, labor informality (2015) is defined as all economic activities that are carried out by workers where there are no formal agreements in place, such as a contract. However, in this study, we conceptualized labor informality as those conditions where workers were not protected by labor legislation; therefore, they did not have social security (Henely and Carneiro, 2009; Contreras and Lacayo, 2020), they lacked access to health services that are provided through formal work, or they did not have a contract where work benefits were established. This conceptualization allowed us to measure the vulnerability of informal workers and their families during the pandemic because they were unable to offset income losses in households, resulting in a more pronounced contraction of informal jobs when compared with formal jobs (ECLAC and ILO, 2021b). Some evidence indicates that there was a 50% drop in the income of informal workers, increased food insecurity, and the depletion of savings (Kesar et al., 2021).

In this study, the term labor informality referred to individuals who did not have access to social security that was provided by formal work or through social programs. Public social security is obtained through formal employment, and social security systems are typically linked to employment and are financed through contributions from employers, employees, or the public sector. In addition, social security systems provide benefits, such as health insurance, retirement benefits, unemployment insurance, occupational hazards coverage, and maternity and paternity leave. Furthermore, according to Salazar (1996) and Chacaltana (2016), informal jobs are those that are not covered by labor legislation and do not contribute to the respective social security of their countries, which implies that they do not have the right to labor justice. Thus, in the literature, access to social security has been used to define labor informality, and, therefore, was employed to characterize labor formality in this study (ILO, 2013; Martínez-Martínez et al., 2022).

The analysis included variables, such as (a) sex; (b) age; (c) whether the respondent was occupied or not during the survey^3^; (d) the expansion factor; and (e) the economic sector where he/she worked. The last variable was recodified according to the International Standard Classification of Occupations to allow for comparability between countries. The economic-sector codes were organized as presented in Table 2.

**Table 2:** Economic sectors (according to ISCO).

Abbreviation	Description
Prim	Primary (Agriculture, livestock, forestry and fishing)
Indm	Mining and quarrying and manufacturing industries
Serv	Supply of basic services (electricity and water)
Cont	Construction and real estate activities
Come	Wholesale and retail trade and auto repair
Hore	Accommodation and food service activities
Trco	Transport and storage, and information and communications
Fini	Financial and insurance activities
Adpu	Public administration and defense
Educ	Teaching and education services
Heal	Human health care and social assistance activities
Oths	Other services

Contreras and Lacayo (2020).

The data were analyzed using several statistical tools. Mostly, the analysis was based on the work of Chacaltana (2016), and the methodology was originally proposed by McMillan and Rodrick (2011). The analysis techniques emphasized the contribution of sectoral change as the determinant of labor formality. The formality rate (*τ*) was calculated using a weighted average between the formal employment rates of the number ( i) of economic sectors ( p ) in a specific period ( t ). Equation 1 represents the statistical model.


(1) $$\tau_{t}=\overset{p}{\underset{i=1}{\sum }}\tau_{i,t}\theta_{i,t}$$

In the equation, $\theta_{i,t}$ is the weight corresponding to the percentage of people that are covered by the social security system. The equation was applied to calculate the informal sector rate for the countries and sexes.

According to Chacaltana (2016), by including the incidence of the sectoral changes, the variation in formality can be broken down into two dimensions, the intrasectoral and the intersectoral components (see Equation 2).


(2) $$\Delta \tau_{q,t}=\overset{p}{\underset{i=1}{\sum }}\theta_{i,q,t-k}\Delta \tau_{i,q,t}+\overset{p}{\underset{i=1}{\sum }}\tau_{i,q,t}\Delta \theta_{i,q,t}\forall \left\{q\in \Omega \right\}$$

The first term, on the right, corresponds to the intrasectoral component, which explains the variation in formality as a product of changes in the sector. The second term, on the left, corresponds to the intersectoral variation and refers to the migration of workers from the informal to the formal sector. Then, ∆ indicates the variation in $\tau_{i}$ and $\theta_{i}$ during the period between t and t−k (see Contreras and Lacayo, 2020).

The study’s unit of analysis was data from individuals over 10 years old.^4^ Additionally, the calculations were disaggregated by sex, and periods were selected according to the following criteria. For the initial period, the first quarter of 2020 or, if unavailable, the earliest period was selected. For the final period, the fourth quarter of 2021, or the last available period, was selected. It is important to note that there were variations in these criteria according to each country and depending on the availability and characteristics of the data. To test the statistical significance of the variations in the weighted formality rates, hypothesis tests of the proportions between the scores for each comparison were conducted (by country; country and gender; and country and economic sector). All statistical calculations were performed with STATA software.

## 3 Results

In this section, the changes in the labor formality rates before and 2 years after the main COVID-19 pandemic contingency measures are described, and these results were analyzed by gender and economic sector to determine the problems in Latin America that need addressing.^5^ Table 3 shows the weighted labor formality rate, i.e., the formality expressed as a percentage of the total employment, in the first quarter of 2020 and the last quarter of 2021 (where applicable) and the variation between both scores. The results indicated that the weighted labor formality rates for the general population increased in almost all countries, except for Ecuador (−4.01 percentage points or p.p.) and Peru (−1.81 p.p.). In contrast, the data from Guatemala and Uruguay reported the highest increment in the region (5.72 p.p. and 2.26 p.p., respectively). Other countries, such as Mexico and Argentina, accounted for slight increases in the formality rate (0.33 p.p. and 0.41 p.p., respectively). All these variation scores between the periods were statistically significant.

**Table 3:** Weighted labor formality rate, by general population.

Country	Weighted labor formality rate			Components	
	2020-Q1	2021-Q4	Variation	Intrasectoral	Intersectoral
Argentina	69.15	69.57	0.41***	−0.07	0.48
Bolivia	21.43	23.25	1.82***	3.03	−1.21
Chile	62.14	63.71	1.57***	1.18	0.38
Colombia	91.01	92.88	1.87***	1.77	0.10
Ecuador	26.46	22.46	−4.01***	−3.24	−0.76
Guatemala	65.93	71.65	5.72***	4.63	1.08
Mexico	40.33	40.66	0.33***	0.34	−0.02
Peru	28.58	26.77	−1.81***	−1.70	−0.11
Uruguay	76.05	78.31	2.26***	1.96	0.30

Source: Authors’ elaboration from multiple datasets. *p < 0.05; **p < 0.01; ***p < 0.001.

Regarding the components of the weighted labor formality rate (the intrasectoral and the intersectoral dimensions), Table 3 shows that, in almost all cases, the intrasectoral scores were higher than the intersectoral components. This suggests that the changes in formality were due to economic sector improvements instead of a shift in the workers from the informal to the formal sector. One exception to this pattern was Argentina, where the intersectoral component score was higher than the intrasectoral score.

The results were also calculated by gender (see Table 4). In men, the weighted labor formality rate was increased in all countries, except for Ecuador and Peru (−3.23 and − 2.04 p.p., respectively). The nations with the highest increases were Bolivia (4.65 p.p.) and Guatemala (4.15 p.p.). These scores were statistically significant. Concerning the components of the formality rate, in all countries, the intrasectoral component scores were the highest in comparison with the intersectoral scores. These numbers suggest that the changes in formality are because of improvements within each economic sector.

**Table 4:** Weighted labor formality rate, by gender.

Country	Weighted labor formality rate			Components	
	2020-Q1	2021-Q4	Variation	Intrasectoral	Intersectoral
Men					
Argentina	66.92	67.76	0.85***	0.46	0.39
Bolivia	24.23	28.88	4.65***	4.42	0.24
Chile	62.83	63.81	0.98***	0.79	0.19
Colombia	89.51	92.09	2.58***	2.46	0.12
Ecuador	26.99	23.76	−3.23***	−2.68	−0.54
Guatemala	65.22	69.37	4.15***	3.40	0.74
Mexico	38.16	38.24	0.09***	0.14	−0.05
Peru	29.45	27.41	−2.04***	−1.65	−0.39
Uruguay	74.78	77.30	2.53***	2.15	0.38
Women					
Argentina	71.96	71.90	−0.06*	−0.89	0.82
Bolivia	18.08	16.96	−1.12***	1.54	−2.66
Chile	61.19	63.57	2.39***	1.86	0.53
Colombia	93.21	94.09	0.89***	0.79	0.10
Ecuador	25.72	20.64	−5.07***	−3.92	−1.15
Guatemala	67.32	75.82	8.50***	5.42	3.08
Mexico	44.18	44.96	0.79***	0.64	0.15
Peru	27.53	25.98	−1.55***	−1.62	0.07
Uruguay	77.59	79.51	1.93***	1.45	0.47

Source: Authors’ elaboration from multiple datasets. *p < 0.05; **p < 0.01; ***p < 0.001.

In the case of women, more countries had a decrease in the weighted labor formality rate: Ecuador (−5.07 p.p.), Peru (−1.55 p.p.), Bolivia (−1.12 p.p.), and Argentina (−0.06 p.p.). Meanwhile, Guatemala (8.50 p.p.) and Chile (2.39 p.p.) were the countries with the highest increase in the formality rate of women. As was the case for men, all the scores were statistically significant. Regarding the components of formality, in almost all situations, the intrasectoral score was higher than the intersectoral score (except for Bolivia). However, it is important to note that when the component score was negative, it indicated a loss of condition. For instance, in Ecuador, both components were negative, suggesting that they had the worst labor conditions and there was a shift in the workers from the formal to informal economic sectors.

**Figure 1:**
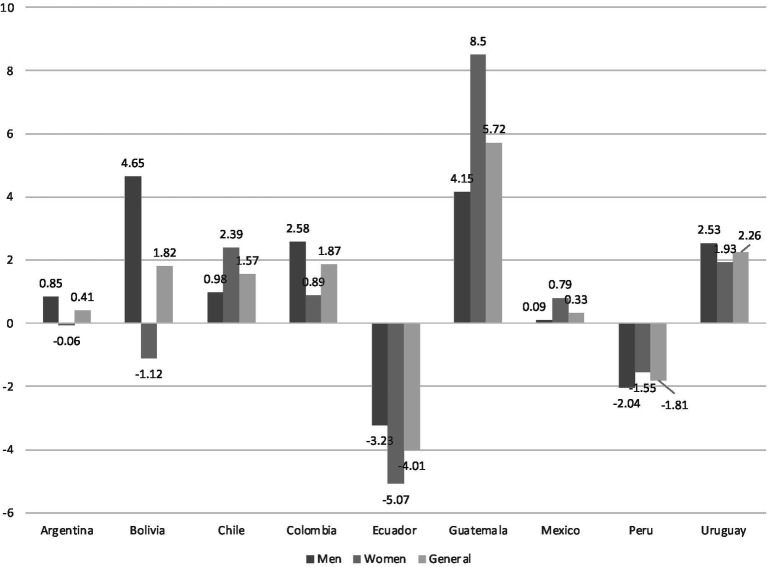
Variations in weighted labor formality rate, by general population and gender. All scores are statistically significant (*p* < 0.05 or less). Source: Authors’ elaboration.

To allow for a comparison between the results, the variations in gender and across the general population are shown in Figure 1.

The weighted labor formality rate was disaggregated by economic sectors, as shown in Table 5. Although there was not a clear pattern of formality between the countries, there was a general tendency of growth (or decline) in formality in some sectors. For instance, on average, the Commerce sector had the highest increment in the region (0.64 p.p.), which was followed by the Transport and Communication (0.33 p.p.), Construction (0.26 p.p.), and Primary sectors (0.22 p.p.). On average, the sectors with the worst performance were the Hotels and Restaurants (−0.32 p.p.), Public Administration (−0.22 p.p.), Other Services (−0.18 p.p.), and Health Services (−0.17 p.p.). As noted in Table 5, most of the variation scores were significant. Interestingly, the Basic Services sector had the fewest significant results among the countries in the study.

**Table 5:** Weighted labor formality rate variation, by sector.

Country	Prim	Indm	Serv	Cont	Come	Hore	Trco	Fini	Adpu	Educ	Heal	Oths
Argentina	−0.02	0.68***	−0.22***	−0.46***	0.67***	−0.10**	0.07*	0.43***	0.00	0.49***	−0.05	−1.09***
Bolivia	0.16***	0.88***	−0.12*	0.36***	1.24***	0.21***	0.40***	−0.10*	−0.70***	−0.91***	−0.51***	0.91***
Chile	−0.32***	0.33***	0.18***	0.16***	0.42***	−0.31***	0.17***	0.14***	0.44***	0.43***	0.41***	−0.49***
Colombia	2.35***	−1.35***	0.00	1.68***	1.23***	−1.01***	0.12***	0.00	0.42***	−0.38***	−0.13***	−1.08***
Ecuador	−0.26***	−0.31***	0.03	−0.32***	−0.65***	−0.29***	−0.07**	0.06	−0.39***	−0.51***	−0.27***	−1.04***
Guatemala	0.46***	1.71***	0.10**	1.39***	2.30***	−0.03	0.59***	−0.11***	−0.02***	−0.11***	0.01	−0.57***
Mexico	0.08***	0.03**	0.03	−0.24***	0.16***	−0.15***	0.05***	0.34***	−0.54***	0.29***	0.22***	0.06***
Peru	−0.50***	0.15***	−0.01	−0.61***	−0.09**	−0.54***	0.85***	−0.19***	−0.84***	0.19***	−0.78***	0.55***
Uruguay	0.02	−0.46***	−0.12	0.40***	0.52***	−0.70***	0.76***	0.23*	−0.31**	1.28***	−0.47***	1.11***
Average	0.22	0.18	−0.01	0.26	0.64	−0.32	0.33	0.09	−0.22	0.09	−0.17	−0.18

Prim, Primary (agriculture, livestock, forestry, and fishing); Indm, Manufacturing industry; Serv, Basic services (electricity and water); Cont, Construction, and property and housing; Come, Commerce, wholesalers and retailers; Hore, Hotels and restaurants; Trco, Transport and communication; Fini, Financial intermediation and related services; Adpu, Public administration; Educ, Teaching and education services; Heal, Health services; Oths, Other services. Source: Authors’ elaboration from multiple datasets. *p < 0.05; **p < 0.01; ***p < 0.001.

There were distinctions in the patterns for each country. For instance, in Argentina, the formality increased by 0.41 p.p. The Manufacturing, Commerce, and Education services increased their formality rate by 0.68, 0.67, and 0.46 p.p., respectively. Whereas the formality rate decreased in the other service, Construction, and Basic service sectors by −1.09, −0.46, and − 0.22 p.p, respectively. In the case of Bolivia, the formality rate improved by 1.82 p.p., which was in the order of Commerce (1.24 p.p.), Other Services (0.91 p.p.), and Industry (0.88 p.p.); whereas Education and Public administration had the worst performance (−0.91 and − 0.70 p.p., respectively). Chile had an increase in formality by 1.57 p.p. The sectors with higher positive variations were Public Administration, Education Services, and Commerce with 0.44, 0.43, and 0.42 p.p. respectively, and the sectors with the highest decreases were Other Services (−0.49 p.p.) and Primary (−0.32 p.p.).

The results also showed that in Colombia there was an increase in the labor formality rate by 1.87 p.p., which was in the order of the Primary sector (2.35 p.p.), Construction (1.68 p.p.), and Commerce (1.23 p.p.). In contrast, the Manufacturing (−1.35 p.p.) and the other service sectors (−1.08 p.p.) reported the highest decrease. Guatemala had a stronger process of formalization during the analyzed period, which was driven by positive variations in several of the economic sectors. In contrast, the Manufacturing and Construction sectors showed increases of 2.30, 1.71, and 1.39 p.p., respectively. In contrast, the other Service (−0.57 p.p.), Education (−0.11 p.p.), and Financial Service sectors (−0.11 p.p.) reported the lowest increase in formality. In the case of Mexico, there was a low improvement in the formality rate (0.33 p.p.), which increased most for the Financial Services, Education Services, and Health Services, by 0.34, 0.29, and 0.22 p.p., respectively. The sectors that reported the highest decreases were the Public Administration (−0.54 p.p.) and Construction sectors (−0.24 p.p.). Regarding Uruguay, the highest increase in formality was in the Education (1.28 p.p.) and Transport and Communication sectors, whereas the highest reduction was in the Hotels and Restaurants sector (−0.70 p.p.).

Finally, as aforementioned, Ecuador and Peru were the two countries with a reduction in formality of −4.01 p.p. and − 1,81 p.p., respectively. In the case of Ecuador, all the sectors, except for Financial and Basic Services, had a decrease in formality, and Other Services had the highest reduction (−1.04 p.p.). In the case of Peru, the reduction was the highest for Public Administration (−0.84 p.p.) and Health Activities (−0.78 p.p.).

## 4 Discussion

In Latin America, on average, the informal sector labor rate was 24.2% in the 2 years before the COVID-19 pandemic started (2018 and 2019; LAPOP, 2020). This was attributed to the recurring economic contractions in the region, which generated losses of formal employment during economic cycles (ILO, 2020). The pandemic had adverse and immediate effects on the labor markets due to sanitary restrictions, such as confinement and social distancing (ECLAC, 2020). This led to an economic crisis and rising unemployment. Hence this study investigated the changes in labor formality rates prior to and 2 years after the main contingency measures of the COVID-19 pandemic were implemented.

According to our findings, the weighted labor formality rate increased in most countries in the region, which was explained by the intrasectoral component. This means that the changes in formality could be attributed to better hiring technology, greater capital accumulation, the integration of productive processes, the integration of marketing processes, and differentiated fiscal stimuli. However, the changes were not due to the displacement of workers from highly informal economic sectors to more formalized sectors (see Martínez-Martínez et al., 2022). The distinctions in the intrasectoral and intersectoral components among the countries May be because of historical trajectories, cultural differences, and economic structures, which shape the dynamics of formal labor markets.

Our findings, contrary to what was expected, suggest that, although the COVID-19 pandemic had devastating effects on the labor market, it also drove certain structural changes within the economic sectors that favored employment formalization. However, the lack of significant movement of workers from the highly informal economic sectors to the more formalized sectors indicates that efforts to improve labor formality have not been effective in all regions.

The literature has shown that during the COVID-19 pandemic, most of the changes in formality were mainly due to the contraction of the informal sector (Martínez‐Martínez and Reyes‐Martínez, 2024; ILO, 2020; Reyes-Martínez and Andrade-Guzmán, 2022). The contraction of employment was more evident in the informal sector than in the formal sector because the confinement measures restricted mobility and informal workers from carrying out tasks that relied on physical proximity to offer services or products and tasks in public spaces. Unlike previous crises, the physical distancing measures limited the ability of the informal sector to act in a countercyclical manner (ECLAC and ILO, 2021b). Additionally, according to the ECLAC and ILO (2021a), the relatively greater impact on informal employment was mainly because formal employment benefited to a larger extent from public policies aimed at safeguarding the labor relationship. These policies encompassed measures, such as job suspensions or reduced working hours, and were accompanied by state subsidies or unemployment benefits in some countries. Consequently, workers in formal employment were able to better withstand the economic repercussions and maintain a certain level of stability, in comparison with those in informal employment. The findings in this study also indicated that Ecuador and Peru had the most adverse conditions in the region because the intrasectoral component decreased, and in both countries, there was almost no displacement of workers who were in the informal sector to the formal sector.

Our analysis of the weighted labor formality rates across the different countries in the region revealed heterogeneous trends across the different economic sectors. Overall, there was a notable increase in the formality rates due to intrasectoral advancements in sectors, such as Manufacturing, Commerce, and Education services. However, this positive trend was counterbalanced by declines in sectors, such as Other Services and Construction. Although a regional trend of increasing labor formality was evident, there were disparities within and between countries, indicating the need for targeted interventions to promote formal employment in various sectors in the region.

According to the ILO (2022a), Ecuador experienced high rates of labor informality and failed to achieve a recovery in formality. This can be attributed to several interrelated factors. First, the country’s social protection system is fragmented and segmented, falling far short of guaranteeing an adequate level of protection in terms of financing, coordination, and governance. The Agriculture, Commerce, and Services sectors were also significantly affected by the COVID-19 pandemic. Additionally, before the pandemic, these sectors exhibited a concentration of employment that was characterized by lower productivity, higher informality, impoverishment, and a lack of protection. Due to the type of employment that was generated by these sectors and the impact of the pandemic, workers in these sectors were further affected, deepening the issue of labor informality in the country. Furthermore, sectors like Manufacturing and Construction, which accounted for a substantial proportion of formal employment prior to the pandemic, suffered significant job losses. Lastly, labor informality was concentrated among groups, such as women, youth, older individuals, and rural residents, reflecting the prevalence of gender roles and the lack of opportunities in rural areas. These factors contributed to Ecuador’s inability to recover formal employment following the pandemic.

In the case of Peru, the lack of improvements in labor formality was attributed to the persistently high rate of labor informality in the country (above 70%), which has been a structural problem since 2017 (Gamero and Pérez, 2020). According to the ILO “Impact of COVID-19 on Employment and Labor Income” report, this situation was exacerbated by a significant decline in GDP and economic activity caused by the COVID-19 pandemic (Gamero and Pérez, 2020). Similar to Ecuador, the sectors of Services, Commerce, and Agriculture, which were characterized by low productivity, were particularly vulnerable, leading to an increase in informality, especially among the less-skilled independent workers. Furthermore, the most affected sectors, such as Trade, Manufacturing, and Accommodation, experienced insufficient income and informal working conditions, further exacerbating the problem (Gamero and Pérez, 2020).

The evidence also indicated that even though several countries in the region returned to the “new normality” in economic activities at different times between 2021 and 2022, the transition from informality to formality was low and even less than that before the pandemic. For this reason, people in the informal sector had the most adverse conditions (ILO, 2020; Khambule, 2020) during confinement and afterward, and Mexico, Brazil, Costa Rica, Paraguay, and the Dominican Republic (ECLAC, 2021) were the countries with the most precarious conditions for this region.

The precariousness of the situation that informal workers experienced during the pandemic created several different problems, such as a significant decrease in income, an increase in food insecurity, and a depletion of savings (Busso et al., 2021; Kesar et al., 2021). Moreover, many workers acquired debts to cover household expenses, because they were not prepared for such a long confinement. For this reason, poor families became even more impoverished (Busso et al., 2021), and much of this was because government interventions were not fully inclusive of informal workers (Khambule, 2020) and because this group previously did not have access to unemployment insurance (Kesar et al., 2021; Pitoyo et al., 2021) in most countries. This means that informal workers were the most affected because they did not have employment guarantees in the event of a crisis that were provided to formal workers (Pitoyo et al., 2020). Thus, we hypothesized that during the COVID-19 pandemic, the informal sector was the most affected and there was a reconfiguration of the traditional mechanism of adjustment to crises through labor informality, unlike in previous crises (McFarlane, 2012; Khambule, 2020). Specifically, the traditional mechanism usually involves labor informality being a source of income for those who were leaving the formal sector and those who were already in it during a crisis. Labor informality usually represents a key mechanism to protect households from the effects of large negative economic shocks (Mapp and Moore, 2015).

The hardest period during the pandemic for people in the informal sector was the period of mandatory confinement in each country. However, due to economic necessity, individuals left confinement to seek work. Not all countries carried out social policy actions that assisted people in crisis, which forced them to leave confinement. For instance, the governments of Belize (4.46%), Brazil (4.02%), Bolivia (2.83%), Peru (2.36%), and Argentina (2.23%) applied the highest percentage of GDP in non-contributory measures, but other countries maintained the same social policies from before the pandemic or they invested very low percentages in extraordinary support measures. Some of these countries in Latin America were Trinidad and Tobago, Mexico, Jamaica, Ecuador, Barbados, the Bahamas, and Uruguay (ECLAC, 2021). There is evidence that the income support programs that were implemented in some countries in Latin America did not fully compensate for the harmful effects of the pandemic (Busso et al., 2021); furthermore, they often did not implement any measures (or only some) for informal workers (Khambule, 2020).

Regarding the results by gender, it is important to contextualize that, before the pandemic, women were more likely to work in informal jobs regardless of individual and household characteristics (Acemoglu, 2001; ILO, 2013). In addition, women in formal jobs were in sectors with lower productivity and low job quality (ILO, 2019; OECD and ILO, 2019); therefore, they were more likely to move from formal economic sectors to informal sectors (Cuevas et al., 2016; Carvajal et al., 2017). These adverse conditions, when added to the effects of the pandemic, can explain the more profound effects on women when compared with men, in the different countries. Generally, the labor reality for women in Latin America is characterized by significant challenges, reflected in the absence of formal contracts, social security, and labor rights. However, in the years preceding the pandemic, improvements were evident in the working conditions for women in specific countries within the region, such as Bolivia, Nicaragua, and Peru, where there was an increase in female participation in the workforce (ECLAC, 2019). Additionally, there was an increase in the average number of years of education for women in Argentina, Colombia, and Uruguay, implying heightened labor expectations and corresponding income increases (Busso and Romero, 2015). In this context, governmental initiatives for labor promotion were implemented in certain countries, as exemplified by Chile. In this nation, the convergence and synergy of various actions in economy, finance, and labor significantly contributed to reducing labor informality, particularly concerning gender-related issues (Henríquez, 2019).

The fact that women are disproportionately affected by informality when compared with their male counterparts is not the only issue. Women were also significantly impacted by a sharp contraction in employment, particularly within the informal sector, due to job losses in sectors, such as tourism and domestic services, which were heavily affected by the crisis. According to Roxana Mauricio’s report on employment and informality in the region for the ILO (2022b), these sectors not only exhibited high informality rates but also demonstrated a feminization of these occupations. This highlights the dual challenge that was faced by women: they were overrepresented in informal employment and disproportionately affected by the economic downturn in sectors that were traditionally dominated by female workers. Addressing these structural issues requires targeted policies aimed at promoting formalization and ensuring gender equality in employment opportunities. However, as mentioned before, the region is heterogeneous, and there were differences between each country.

In the cases of Ecuador and Peru, both men and women did not have increases in formality; therefore, the conditions were adverse regardless of gender. In most countries, men experienced higher rates of weighted labor formality when compared with women. This means that women had not transitioned from informality to formality before the pandemic, and more than 2 years after it began, they had the most layoffs due to the sectors in which they worked (ECLAC and ILO, 2021). Our findings emphasize the precarious situation of many women in the region, regardless of the country, but most strongly in Ecuador, Peru, Bolivia, and Argentina. The ECLAC (2021) stated that the pandemic crisis generated a setback, further widening the inequality gap in labor occupation by gender, where women were the most affected. These conditions amplified the income gaps between men and women, which, in turn, restricted the possibility of increasing women’s incomes and limited their economic autonomy (UNU and ECLAC, 2020). Additionally, during the pandemic and in confinement, there was an increase in unpaid care, due to the closure of schools and daycare centers (Llanes and Pacheco, 2021; Zamudio et al., 2022). Other components that May have affected results by gender, such as the role of gender roles, stereotypes, and behaviors, were not captured in this study. These aspects May have influenced different outcomes in formality by gender across these countries and sectors. Therefore, efforts should be made to provide support and training programs tailored to women in the workforce, particularly those in informal sectors that are heavily impacted by economic crises.

Regarding the economic sectors, the containment measures that were carried out in each country negatively affected the labor market because production and consumption activities were interrupted in various sectors. Although these measures affected each nation differently, there was a clear trend in most of them. The most affected sectors were those that were categorized as non-essential by governments, especially those that were related to services, such as hotels and restaurants, public administration, and other services. The loss of formality in the service, hotel, and restaurant sectors was clearly due to the contingency and social isolation measures throughout the region, which restricted free movement and public activity. The effect on labor formality in the public administration sector could be because of the operational impact on public institutions, which resulted in a reduction in hiring and the suspension of new entries into the public sector.

Notably, despite the healthcare sector being considered an essential sector, especially within the context of the pandemic, it also experienced issues of labor informality. According to the ILO (2022b), COVID-19 generated changes in the employment conditions of healthcare workers, including a decrease in income, an increase in working hours and appropriate employment, and an increase in the hiring of independent workers rather than salaried employees. These conditions can be attributed to the increased healthcare needs, the rise in demand for certain healthcare professionals, the reduction of non-COVID-19 related healthcare services, and changes in work schedules.

In contrast, several sectors reported, on average, increases in formality. These economic sectors were those that were considered, by most countries, to be essential in the production and supply chain, such as the commerce, primary, construction, transportation, and communications sectors. In almost all countries there were more increases in the formal economic sectors, except for Peru and Ecuador, where almost all the economic sectors had a reduction in formality. However, improvements in the conditions of each sector could be a secondary effect of job losses in the informal economy during the COVID-19 pandemic (Pitoyo et al., 2020, 2021). Therefore, we cannot attribute the growth of formality to a single cause, and each sector needs to be investigated separately to understand the specific dynamics.

## 5 Conclusion

In the history of the contemporary world, one of the greatest global problems was the COVID-19 pandemic. Latin America experienced repercussions on the labor markets, with certain characteristics that were not seen during previous crises. Notably, labor informality did not assist with maintaining household incomes during economic difficulties because it ceased to function as a protective mechanism for people who were left without a job in the formal sector or who were already in these types of jobs. This could mean that informality was not countercyclical during this COVID-19 pandemic, especially during the period of confinement in each country.

Although the first 2 years post-pandemic showed some signs of recovery, most countries’ economies have still not returned to pre-pandemic levels, where the rate of labor informality was high, 90% on average in developing countries, 67% in emerging countries, and 18% in developed economies (ILO, 2018). Furthermore, the structure of the labor market in Latin America shows that the labor formality rate (access to social security via formal work) continues to be very low in a labor market with little structural mobility and limited absorption capacity.

The evidence found in the study also agrees with other research carried out in Peru (Roncal, 2022) and Mexico (Zamudio et al., 2022) regarding the negative effects that the pandemic had on labor markets, especially on informal employment, in addition to having greater repercussions on women, making gender inequalities greater than those that existed before the pandemic. Likewise, other research in Latin American countries that were not part of the study, such as Venezuela (Marotta and Ponce, 2022), where they find that the COVID-19 pandemic caused a deterioration in the labor market, especially in informal employment. In the case of El Salvador, as in our study, they find that since the beginning of the pandemic, people in the formal market were the least affected. In this study (Barrera, 2022) they point out that although some people suffered a reduction in their salaries, they did not lose their jobs, and several switched to teleworking.

The lack of robust social policies in Latin America mostly affected people in labor informality. Although these policies do not lift people out of poverty, implementation of income support programs could have helped to prevent pre-existing inequalities in the region, since programs of this type function as a protective barrier against economic crises (Martínez-Martínez et al., 2020), such as the COVID-19 pandemic. Based on the findings of this study, there is a need to design public policies that reverse the current situation of the labor market and prevent future economic shocks, with special emphasis on the informal sector. Therefore, a social protection system could ensure a basic income, mostly for people in poverty, or unemployment insurance could be provided, regardless of employment status (formal or informal). There is also a need for a support system for businesses, especially those that were not a priority for the government because they were not selected as essential, including small businesses (formal and informal).

According to Gálvez Muñoz and Rodríguez Madroño (2011), economic crises tend to worsen women’s working conditions, which results in lower wages, more precarious jobs, and a higher incidence of informal employment or unstable part-time jobs. The recovery of female employment tends to lag behind that of male employment, due to public policies that often favor male employment because men are seen as the primary providers for families (Gálvez Muñoz and Rodríguez Madroño, 2011). Therefore, it is essential to consider the entrenched gender inequalities in the labor market when designing economic recovery policies. The inequalities are a clear characteristic of this region. However, by acknowledging the unique socio-economic disparities within and among the countries, policymakers could focus on transformation for the development model in this region. Specific measures could be implemented that address both economic factors and gender roles in our society. For example, ensuring equal job opportunities, closing the gender wage gap, and supporting childcare programs to promote better work-life balance. These actions can lead to a fairer and more sustainable recovery, strengthening economic resilience against future crises.

Given the unprecedented nature of the pandemic globally, it resulted in the halting of production and economic activity. Throughout this study, we observed variations in the impact of the pandemic that were influenced by productivity levels, gender disparities, and sectoral differences. Countries have had to implement various measures to mitigate both health and economic impacts. According to the UNU and ECLAC (2020), a key policy response should be universal social protection, as it has the potential to reduce inequalities, promote progress toward social inclusion, and foster inclusive growth. The COVID-19 pandemic generated new challenges in the short and medium term, due to the economic crisis. Moreover, the traditional mechanism based on previous crises differed during the pandemic due to the behavior of the labor market, which resulted in new challenges for governments that had to respond promptly. To date, the social and economic effects of this pandemic have not been fully realized even though it officially ended in May of 2023; however, as social and economic impacts continue to unfold, there is a need for adaptive responses.

It is important to recognize some limitations of the study. First, when carrying out comparative research there is always the risk of generalizing the circumstances of the countries when they experienced adverse conditions in different ways during the pandemic. Second, this study focused on the composition of employment changes but did not consider whether this May be due to changes in labor participation during the pandemic, as this was beyond the scope of our research. Third, regarding the methodology, one of the problems was the standardization of the information in the different countries of the study. Although in most cases the data was available every quarter, in others, it was not, so the information closest to the study period was used. Despite these limitations, the study had a strong methodology that was reflected by the clear trend in the data of the region.

## Data Availability

Publicly available datasets were analyzed in this study. These data can be found on the websites of the Statistical Institutes specified in Table 1.

## Footnotes

^1^During the research process, the datasets from some countries were not available because of technical issues, some government associations did not respond to requests for datasets, or the data were not made available to the public.

^2^Typically, household surveys do not rely on activity sectors as a reference for sample construction. As a result, labor market analysis at the sectoral level May suffer from underrepresentation or overrepresentation of certain sectors relative to others.

^3^Occupied included subordinate and remunerated, self-employed, and underemployed individuals, all economically active population. It does not include those economically active population that is not occupied.

^4^According to the United Nations International Children’s Emergency Fund (UNICEF, n.d.), in Latin America, the minimum age for employment ranges from 14 to 18 years of age. However, many individuals below that age enter the job market due to economic conditions, and most of them belong to the informal sector (Salazar, 1996). Thus, most national statistics in the region collect data for child labor.

^5^It is crucial to recognize that economic activity is influenced by seasonal variations each year. Consequently, although we conducted a comparison between the first and fourth quarters to examine developments at the beginning and end of a significant period, these results must be interpreted with careful consideration of the seasonal context.
